# The neural antecedents to voluntary action: Response to commentaries

**DOI:** 10.1080/17588928.2015.1054271

**Published:** 2015-06-16

**Authors:** Parashkev Nachev, Peter Hacker

**Affiliations:** ^a^ Institute of Neurology, UCL, London, UK; ^b^ Department of Philosophy, University of Kent, Kent, UK

**Keywords:** Voluntary action, Conceptual analysis, Readiness potential, Neurology of law

## Abstract

Cognitive neuroscience must attend to the conceptual coherence of its hypotheses as well as to their empirical support. Examining the most influential studies of the neural antecedents to voluntary action, our Discussion Paper sought to identify the real-world consequences of neglecting the former in what we argued has been too narrow a pursuit of the latter. Though conceptual in form, our analysis is sharply empirical in its conclusions, revealing what have long been thought to be momentous experimental observations—such as the readiness potential—as the outcome of previously unidentified confounds that rob them of significance. Conversely, we suggested that experimental studies of two-way control, amongst other defining features of the voluntary, have been given less emphasis than the subject demands. Here, we ramify our analysis down the paths identified by others in the commentaries we received.

The neural basis of voluntary action is arguably as cardinal a problem in behavioral neuroscience as any. It certainly cannot be said to be a peripheral, niche interest, nor one lacking in intelligent attention over at least a century of intense study. If neuroscience is a mature discipline, then the study of voluntary action ought to be mature too, comfortably settled in its fundamental conceptual framework, clear in the definition and stratification of its constituent problems. Whatever the merits and demerits of our treatment of just one aspect—the neural antecedents to action—that it should elicit commentaries so diverse in their range and point is surely evidence of a far greater need of conceptual evaluation than the surface historical record suggests. For if the many terabytes of experimental data gathered in the field are balanced on so small an agreed common conceptual ground, the structure cannot remain upright for very long. It is easy to forget that the empirical rests on the conceptual, and so cannot grow without it.

That need, it must be admitted, is not easy to satisfy, for the form of analysis it requires is hard to practice and even harder to teach. This is not so much because it is intellectually taxing as because it is not merely a matter of mechanically applying any specifiable set of general principles. The best one can do is to *illustrate* the approach, in concrete examples, as we do below in loosely structured responses to the diversity of commentaries we received.

## THE CONCEPTUAL AND THE EMPIRICAL

Ramey and Chrysikou are correct that the conceptual and the empirical are distinct (Ramey & Chrysikou, 2014). A conceptual error cannot be corrected by empirical effort, and vice versa. But the two are not ships that pass in the night, neither bound for the destination of the other. Each plays its part in the joint enterprise of revealing the true nature of human beings, and both need to be right if the enterprise is to succeed. Moreover, while it is true that senseless ideas are often more pernicious than falsehoods, neither is benign. Obtaining experimental data—at least in behavioral neuroscience—is not like quarrying marble, of value regardless of the architecture the material is used to build, because one may always reuse it in another design. Here the data are derived from specific, often highly contrived, behavioral paradigms that make the general applicability conditional on the validity of the underlying hypothesis. Where the hypothesis is invalid, and the data have a trivial explanation such as temporal affordance in the case of the *Bereitschaftpotential*, the empirical effort cannot be of much use to anyone.

Inconsequential data, furthermore, make it harder to resist the temptation to allow the horizon of empirical possibilities—inevitably constrained by the technology at our disposal—to limit the space of conceptual possibilities. It is true that there is little point in talking about hypotheses for which there is no empirical test to hand. But equally there are no grounds for expecting the correct hypothesis to be easily testable. In science, the question of whether or not the data fit a hypothesis matters *only* when the space of possible hypotheses is reasonably defined. If the facts admit thousands of other hypotheses one has not even considered, the only “empirical support” they can provide is to the experimenter’s vanity. It is like being pleased with one’s horse coming first in a race in which the rest of the field has not even entered. Where the subject of study is as complex as the brain, adequate hypothesis comparison is obviously very hard to achieve. But then one’s claims to knowledge should be appropriately tempered: We should admit that there are things we *cannot* know, at least not with the technology at our disposal. If this is obscurantism, it is virtuous obscurantism.

Now it may well be, as Ramey and Chrysikou suggest, that new empirical methodologies such as multivariate fMRI will change this state of affairs. High-dimensional multivariate approaches can certainly demonstrate how misguided attempts to short-circuit the complexity of the brain can lead to errors, and how such errors may remain undetected for decades (Mah, Husain, Rees, & Nachev, 2014). But we do not need to wait for advances in experimental methodology to get our conceptions right: All it needs is careful thought about the constituent concepts, and none of us has any excuse to forego it.

Allowing oneself to be swayed by the empirical exposes one to another danger: The misapplication to the biological, by mistaken analogy, of concepts properly only applicable to the methodological. The key example here is the concept of *noise*. The measurement of any quantity in the real world is inevitably limited by inaccuracies in the measuring tool, whatever that might be. One can never hold it against an experimental result that it is not perfectly accurate, for the opposite would be perfectly implausible. Where a set of observations differs from a hypothesized theoretical relation it *may* still be reasonable to believe that the relation holds. But this is true only if the residual, unexplained variance in the observations *is not open to a better explanation*. In statistical terms this is conceived in terms of lack of structure in the residual error, though, of course, that a structure is not evident at a given resolution of parameterization does not mean that a richer approach could not identify one. But in the domain of voluntary action there is nearly always a better explanation: The subject’s avowal or report of what he did or meant to do. The avowal or report of what the subject did or meant to do is a logical criterion for the voluntariness and intentionality of his deed. It is, to be sure, defeasible, but if undefeated, it provides logically good evidence for the character of the deed. We have shown that neurophysiological predictions from Libet-style experiments not only do not have sufficient specificity for the chosen action but also do not make a comparison with the subject’s ability to predict *at the relevant time* his own intentional actions, assuming unjustifiably, that his prediction is then no better than a guess. The identification of probabilistic biases Sumner discusses at length therefore has no implications for the locus of control, as he says, for the marginal uncertainty is not noise superimposed on a deterministic relation thereby revealed, but the natural variability of mere neural correlates that need have no mechanistic—let alone deterministic—significance (Sumner, 2014).

To say this is not to say that a set of physiological parameters could not be shown to predict an action, or that a perfectly causal account of a set of voluntary movements is conceptually impossible: We do not have to go that far because the data fall so short. Human beings are biological creatures: Their thought and behavior are dependent on physical and chemical processes. That the causal explanations of voluntary movements that we have been criticizing are incoherent does not imply that others must be too. All we are insisting on here is the need for conceptual fidelity in *stating* hypotheses, just as good scientists naturally insist on empirical fidelity in *testing* hypotheses, and the hypotheses offered here have been found wanting.

That process, which might be called hypothesis clarification, implies a careful evaluation of the constituent concepts of a hypothesis. Just as the well-formedness of a mathematical formula needs to be established by examining the relations of its constituent terms in algebra, so the intelligibility of a scientific hypothesis needs to be established by examining the relations of its constituent terms in logical grammar. That logical grammar is vastly more complex than algebra does not mean one can ignore its rules, for it is they that determine what does and what does not make sense. A condition for a hypothesis to be either true or false is that it makes sense. Moreover, the rules cannot arbitrarily be changed *without exploring the ramifying consequences*, just as the substitution of imaginary for real numbers cannot be done without altering their algebraic relations.

Schurger’s skepticism about such conceptual analysis as a distinct mode of thought is understandable: After all, the approach is hard to specify in terms of explicit rules, technicalities, helpful generalizations: The natural intellectual equipment of academia (Schurger, 2014). Indeed, it is precisely these characteristics of the academic that often land one in deep water, in need of the rope only conceptual analysis can throw. For, as we have argued, the principal difficulties arise not because the solutions are hard but because the problems are *opaque* and further compounded by the usual intellectual apparatus brought to bear on them. For every biological fact that is illuminated by a novel analogy, an extension to the use of an established concept, or some other conceptual innovation, a dozen are thrown into greater darkness. The only way to proceed is step-by-step, testing the ground along the way, trusting least the paths all others have followed.

Be that as it may, that conceptual analysis is desperately needed seems to us compellingly illustrated by the case of the *Bereitschaftspotential*. Considered one of the seminal findings in neurophysiology for over half a century, referred to in more than 6000 scientific books and articles, the physiological foundation of the Libet paradigm—the most influential behavioral paradigm in the study of voluntary action—it is now shown to be merely a temporal variant of the well-known phenomenon of affordance. That inference does not proceed from any new experimental effort—though it may be reflected in it, as it is in Schurger’s own insightful recent work (Schurger, Sitt, & Dehaene, 2012)—but purely from a careful examination of the constituent concepts. The vast majority of neuroscientists have no difficulty in identifying task affordance as a confound, for the individuation of actions in terms of their mechanics and objects is perfectly natural and familiar. The failure to identify temporal affordance here comes from the failure to apprehend the individuation of actions in terms of their *timing* (von Wright, 1963). Once that is recognized it is easy to see that the *absence* of temporal affordance would be profoundly surprising, for it would imply that the brain *must* respond to temporal possibilities differently from any other feature of an action, which is obviously unwarranted, and would go against one of the few empirical certainties about the brain: That it is anticipatory in its activity. That we happen to treat the temporal individuation of actions differently in language is no grounds for assuming the underlying neural activity must follow suit. It is something to be shown experimentally, a prerequisite for the use of the *Bereitschaftspotential* as the foundation of edifices that are otherwise fit only for demolition practice.

## BEYOND THE ANTECEDENTS

A comprehensive conceptual investigation into voluntary action would be a book, not a paper. In confining ourselves to the antecedents here we do not wish to imply contemporaneous or subsequent neural aspects are immaterial. On the contrary, we wish to *dissolve* what we argue is mostly incoherent speculation about the antecedents precisely so as to shift attention to the later aspects Schall and Garr discuss (Schall & Garr, 2014). That this is necessary is easily seen from the disproportionate space discussion of the antecedents takes up in the literature: The most cited papers are either Libet’s or employ some variant of his flawed behavioral paradigm.

Since one usually acts so as to affect a desired change in the environment, voluntary actions that are also intentional have a goal, unless they are done intentionally out of mere inclination (without any further purpose). They may, or may not, achieve their goal. Such actions, moreover, are *learned*, which necessarily implies adaptation driven by the mismatch between desired and achieved outcome, optimized over time. No comprehensive account of voluntary and intentional action could therefore omit the consequences of acting: This is conceptually given.

The reflection of teleological aspects in the neural substrate, however, is not easily investigated empirically. Within a simple conative behavior such as seeking the satisfaction of thirst, causally relating the goal to the behavior *may* be relatively straightforward. One might identify a simple biological parameter, say serum osmolarity, that is intelligibly conceived as the cause of a subsequent act of drinking. But it is hard to see how the goal of avoiding rush-hour congestion by taking the 4.15 train to Cambridge rather than the 5.15 can be built into a causal account of the action of changing one’s itinerary. We have developed the manifold forms of explanation of action, including making sense of the teleological in terms of *reasons*, driven by a need for understanding that simple causal accounts cannot meet (see Hacker, 2007, p. 219). But that practice has emerged for—and therefore need only satisfy—the purpose of making our actions intelligible to each other. It is, however, unsuited to the purpose of explaining the neural underpinnings of voluntary action or any other human power, for that is far from the driving force of its development. *If* we are to make sense of the teleological in neural terms, reasons must be part of the explanandum, not of the explanation. It is far from clear what that might imply, since reasons for acting are not causes but warrants or purported warrants.

The notion of goals naturally presupposes a way of selecting one goal over another. This “executive” aspect, rightly emphasized by Schall and Garr (2014), is constitutive of two-way control, and therefore a feature of *all* intentional actions, not only complex ones, indeed, of all that can bear the name of action, as opposed to mere bodily movement. When a subject in a psychophysical experiment is engaged in something simple, say horizontal saccades in response to arrow cues at fixation, “executive control” is not suspended, for he may at any time do something else of arbitrary complexity. The *compartmentalization* of the notion of the executive, so widespread in the literature (e.g., Norman & Shallice, 1986), as something that comes into play only occurrently, is faithless to the fundamental nature of voluntary action. It is also incoherent, for it implies a mechanism for invoking the executive, a “super-executive,” that is vulnerable to the same problem, thereby setting up an infinite regress. The influential notion of a conflict detector (Botvinick, Braver, Barch, Carter, & Cohen, 2001) does not help here, for the degree of compatibility or similarity between two movements is orthogonal to the relative desirability of the goals they are intended to secure. No simple index could plausibly determine what is at stake at any point on the horizon of action; the powers this requires are too numerous and complex to be assigned to any isolated region of the brain, let alone a process. The impulse to do this needs to be recognized as the crypto-Cartesianism it can only be (see Hacker & Bennett, 2003 for a comprehensive survey, and Nachev, 2011 for discussion in relation to the notion of executive control).

## THE CONTINGENT AUTOMATON

Ben-Yami argues that we overestimate the vulnerability of voluntary action to neural redefinition (Ben-Yami, 2014). One naturally distinguishes the voluntary from the involuntary without inspecting the agent’s brain. The distinction relies on cognitive and behavioral criteria: All that examination of the neurophysiology can do is to clarify their reflection in the neural substrate, not to redefine them. Indeed, the attribution of any neurophysiological feature *presupposes* a prior behavioral categorization, for that is what individuates it in the first place. A neurophysiological feature common to both kinds of action would be properly interpreted as non-specific, not as grounds for reclassifying the actions it accompanies. Without a fully deterministic account of the brain, the behavior is always the dog, the neurophysiology always the tail.

But although the general criteria are clear, their *application* to any specific instance of behavior need not be. Distinguishing between voluntary and involuntary movements in pathological conditions can be very difficult. Patients with complex partial seizures, for example, may exhibit movements that appear sufficiently voluntary to confuse even experienced neurologists. The drug-seeking behavior of an addict may *look* voluntary—inevitably construed on the model of normal conative behaviors where no compulsion is ever truly irresistible—but it is perfectly possible to conceive of a drug that reinforces its taking absolutely, turning the victim into a drug-seeking automaton. Neurophysiology cannot change the criteria of voluntary action, but it may help us apply them correctly where the surface picture is unclear or misleading.

Two aspects of the distinction between the voluntary and the involuntary interact to cause this difficulty. First, the most important criterion of voluntariness—two-way control—is essentially counterfactual. One-way control is easily established by asking someone to *do* something. But to test that someone is able *not* to do something inevitably presupposes a set of *conditions* under which one *would* do it (if it is indeed involuntary), otherwise it makes little sense to speak of anything being withheld. To establish the presence of two-way control one therefore has to replicate the conditions that prompt the movement, not the movement itself. Second, both the *nature* and *timing* of the conditions may be opaque enough for it to be far from obvious what is to be replicated. Where the behavior has an identifiable object, as with conative behaviors, this may be relatively easy: Handing an alcoholic a glass of wine, say, and counting the number of occasions he is able to stop himself from drinking it over some fixed interval of time. But even then, the critical conditions *need not include the object itself* (e.g., “needle freak” drug addicts who become collaterally addicted to the act of injection alone). Furthermore, the *temporal* relation between the conditions and the behavior may be opaque: Not only need they not be time-locked, the former could be arbitrarily antecedent to the latter. The sight of a glass of wine may compel someone irresistibly to drain it *only* when he has been exposed to some other sensory association earlier in the day. Since the critical conditions need make themselves manifest *only* in the observed behavior—what we are using to identify them in the first place—it may be exceedingly difficult to get them into clear view. What matters most is not *what* the agent does but what he does *not* do, in circumstances neither the nature nor the timing of the movement need illuminate. It is therefore perfectly possible for someone to lack two-way control over an action without anyone noticing: Including the agent himself. Such covert automaticity cannot be common, for we generally change our actions—both spontaneously and in response to changes in the environment—too flexibly for it to be anything but an exception, yet it may well occur.

Where a hidden set of critical conditions is invariantly associated with an action, then, identifying a predictive neurophysiological antecedent *may* help us establish the presence of covert automaticity. The facility of this depends on the locus of the conditions. Where they are external to the agent, beyond the horizon of his control, automaticity is easy to establish for we can show that we can *control* him simply by recreating the conditions. By contrast, where they are confined to the body of the agent, we can only *predict* what he will do (assuming that we have no control over his body), and even then predict only as early as the antecedent appears, which as we have seen is usually measured only in milliseconds. Prediction alone does not imply automaticity, it merely opens a temporal interval (between the antecedent and the action) during which one can test for the absence of two-way control. But since the characteristic flexibility of voluntary action typically operates at time scales much longer than milliseconds, the timing of observed predictive antecedents leaves no room. In such circumstances the question of covert automaticity is therefore constitutively indeterminable: We plainly cannot test two-way control if we cannot dissociate an action from the conditions under which it is supposed to be withheld.

To make so much of the exceptional (and the pathological) may be thought unhelpfully to shift the focus away from the general nature of voluntary action, which is the primary concern. But what a synoptic survey of the conceptual landscape here lacks is precisely the hidden, dark ravines between the bright peaks in clear view. It is there that one is prone to lose one’s footing, and is most in need of a sharp conceptual icepick to stay on the track. Moreover, pathology surrounds (and eventually engulfs) all of us, blending with normality at multiple points in the domain of action of considerable social significance.

In illustration, imagine the hypothetical case of a “contingent automaton”: A man addicted to a harmful drug to the extent of being completely unable to resist taking it under some specific set of environmental conditions. Where these conditions obtain, he will *always* take the drug—involuntarily—where they do not, he may or may not take it—voluntarily. In ignorance of the identity of the critical conditions his drug-seeking behavior is most naturally described as the outcome of an imperfect two-way power: Always being able to choose to take the drug, but only sometimes succeeding in resisting it. So perceived, the case mirrors conventional conative behavior where the agent may be said occasionally to have yielded to a temptation, and is correctly condemned—morally—for failing to resist it because he *could* have done otherwise. But the rules of normal conative behavior do not apply where the set of critical conditions is met. Then the behavior is involuntary, and is no more deserving of opprobrium than a knee-jerk or anything else comparably automatic.

Now the critical conditions may be far from obvious, as we have said, and their apparent absence is no grounds for dismissing the possibility. Just as an action is voluntary if the behavioral criteria of voluntariness are satisfied, so it is *not* voluntary if they are not, no matter how complex or elaborate its behavioral and cognitive features. Equally, our contingent automaton may tell us that he *could* have done otherwise, that he *will* resist next time, that *this* was an exceptional case, and so on, but his avowal, though criterial (logically good evidence) for his intentional action, can be defeated by what he actually does. If we are able to identify a set of critical conditions that make the action automatic—with or without the help of a neurophysiological marker—then the normal conative game is no longer in play, whatever the agent might say. Of course, this is only a hypothetical case that may or may not ever obtain in reality. Our task is to mark the limits of conceptual possibility, not to make any claims about the fundamental nature of addiction, which is an empirical matter to be determined by clinical study.

## MORALITY AND THE LAW

The domain of action is partly coincident with the domains of morality and law. A change in our conception of the fundamental nature of action, whether through neuroscience or other means, will naturally ramify into its moral and legal contexts. This is especially true of a change that affects the applicability of the notion of responsibility, for on that rests the key distinction between an agent who may be condemned or praised for his actions and a patient whose actions may only evoke sympathy. Where the distinction is clear—in the obviously insane—the moral and legal positions are largely settled. The boundaries, however, are a matter of contention, over a wide territory.

The difficulty here reflects the practical obstacles of establishing two-way control. That crucial criterion is not at issue, only its application. It is widely agreed that establishing an invariant relation between a set of conditions not under the control of the agent and an action would be grounds for claiming automaticity, for absence of two-way power is thereby implied. The common mistake—as Patterson points out—is to assert that this follows *merely* from identifying a neural antecedent (Patterson, 2014). To argue thus is to misunderstand both the neuroscience and its conceptual context. A neural antecedent may only be a *preliminary* to a test of two-way control, and even then only when it is empirically shown to be *specific* to the action in question, a feat yet to be accomplished outside the obviously pathological domain. Not only has no test of two-way control ever been carried out for a reasonably complex action, no one has ever identified a sufficiently specific antecedent. We are at least twice removed from being able to *begin* to test the assumptions on which thinkers such as those Patterson criticizes are attempting to build complex legal structures. It is not quicksand they are building on, it is thin air.

To say this is not to say that neurology is irrelevant to the law. If it is relevant to obviously pathological cases—and there is no doubt about that—it will be relevant to those falling across the shadowlines of normality: There are no grounds for a sharp boundary. As we naturally make allowance in our moral judgments for obvious bodily defects that may limit someone’s powers of action *physically*—e.g., being prevented from chasing a thief by a limp—so we should make allowance for covert neural defects that limit an agent’s powers of action *morally*. There is universal agreement that the power to run has a somatic dependent; it would be very odd to insist that the power to act morally does not. The neurological literature is in any event littered with illustrative cases of selective moral defects demonstrably caused by focal brain dysfunction (and reversed by its remedy).

Unlike obvious physical defects, however, where it often makes little sense to doubt the loss of a dependent power, neural defects need to be inductively associated with the power in question in the first place. Where the defect is not reversible—as is very common in neurology—the inductive association will inevitably depend on the study of *other* patients, and therefore on the degree of anatomical-functional variability in the brain across the population. Since this is high, especially for the regions of the brain on which complex thought and behavior depend, the association will rarely be strong except in cases where the pathology is gross. But in such cases the manifest behavior is usually sufficient evidence. In any event, the most neurology can do here is to *clarify* the observed behavior, not to overrule it.

## HYPNOSIS

The human brain is easily the most complex biological entity we know (or, rather, do not know). We have neither the investigative technology nor the computational power to characterize and model it comprehensively. Such models as we can build are therefore necessarily partial, removing from the scene the myriad aspects outside our technical or intellectual reach. Indeed, this is how cognitive neuroscience experiments—especially in imaging—are commonly conceived. The experimenter will typically identify two neural states supposedly differing *only* along a single, tractable dimension, the contrast between them assuming to yield the relation of the activity of the neural substrate to the dimension of interest. That there is a great deal here that is unknown therefore seems not to matter, for the unknown is on both sides of the contrast. We divide by it, so to speak, leaving its actuality immaterial.

The problem with the unknown here, however, is that its *interaction* with the dimension of interest is also, by definition, unknown. The degree to which such interactions may confound our contrast is therefore unknown. It may well be, as we have shown for the *Bereitschaftspotential*, that a plausibly invalidating interaction will be found. To say this is not to be nihilist about neuroscience, but rather to emphasize the importance of being on the sharp lookout for confounding interactions when one operates in so much uncertainty.

What we definitely cannot do, however, is to remove interactions simply by closing our eyes to them. And that is what is implied in the use of hypnosis as a means of dissociating an action from its reported voluntariness (Lanfranco, Adolfi, & Ibáñez, 2014). It is constitutive of hypnosis that the subject is in a state that differs from normality in ways *other* than the specific aspect the hypnotist chooses to modify: That is why we speak of someone being *under* hypnosis. Were it not so, we would not speak of hypnosis as a state at all. Someone hypnotically misreporting his voluntary actions as involuntary (or, for that matter, belonging to God, the Devil, or anyone else) is not changed merely in that respect. Indeed, hypnosis proceeds by making someone *receptive* to suggestion and *only then inducing it*. That first, wholly opaque, step makes all subsequent ones uninterpretable. An action may be intelligibly rendered involuntary by hypnosis no more than it may be thereby intelligibly rendered divine.

What hypnosis does illustrate, however, is that a subject’s avowal of or report on the voluntariness or intentionality of his movement is always defeasible. As so often in life, what matters most is what one *does*, not what one *says*. Indeed, a single avowal or report could never be definitive here, for the presence or absence of voluntariness—where it is in doubt—requires the evaluation of two-way control. There are many ways of interfering with the subject’s report, but as long as the power to do otherwise is retained the action is still voluntary.

No potential conflict of interest was reported by the authors.
